# Revisiting the Impact of Neurodegenerative Proteins in Epilepsy: Focus on Alpha-Synuclein, Beta-Amyloid, and Tau

**DOI:** 10.3390/biology9060122

**Published:** 2020-06-12

**Authors:** Yam Nath Paudel, Efthalia Angelopoulou, Christina Piperi, Iekhsan Othman, Mohd. Farooq Shaikh

**Affiliations:** 1 Neuropharmacology Research Strength, Jeffrey Cheah School of Medicine and Health Sciences, Monash University Malaysia, Bandar Sunway, Selangor 47500, Malaysia; yam.paudel@monash.edu (Y.N.P.); Iekhsan.othman@monash.edu (I.O.); 2 Department of Biological Chemistry, Medical School, National and Kapodistrian University of Athens, 75 M. Asias Street, 11527 Athens, Greece; angelthal@med.uoa.gr

**Keywords:** neurodegeneration, epileptogenesis, alpha-synuclein, Aβ, tau

## Abstract

Lack of disease-modifying therapy against epileptogenesis reflects the complexity of the disease pathogenesis as well as the high demand to explore novel treatment strategies. In the pursuit of developing new therapeutic strategies against epileptogenesis, neurodegenerative proteins have recently gained increased attention. Owing to the fact that neurodegenerative disease and epileptogenesis possibly share a common underlying mechanism, targeting neurodegenerative proteins against epileptogenesis might represent a promising therapeutic approach. Herein, we review the association of neurodegenerative proteins, such as α-synuclein, amyloid-beta (Aβ), and tau protein, with epilepsy. Providing insight into the α-synuclein, Aβ and tau protein-mediated neurodegeneration mechanisms, and their implication in epileptogenesis will pave the way towards the development of new agents and treatment strategies.

## 1. Introduction

Epileptogenesis refers to the gradual process by which the normal brain develops epilepsy. It involves an early brain-damaging insult, which prompts a cascade of molecular and cellular alterations that might ultimately lead to the occurrence of spontaneous seizures [1]. Epilepsy is a devastating brain disorder exhibited by an enduring susceptibility to induce epileptic seizures [2]. It is characterized by the occurrence (more than 24 h apart) of two unprovoked seizures, a single unprovoked seizure with a high risk of relapse, and the appearance of epileptic syndrome [3]. Epilepsy is caused by abnormal coordinated firing of neuronal cells mainly due to disparity among excitatory and inhibitory neurotransmission [4]. Epilepsy has emerged as a serious global health concern affection around 70 million individuals of the population worldwide [5]. There is an increased understanding that epilepsy does not merely exist alone, and it is always associated with other neurobehavioral comorbidities, including cognitive impairment, depression, anxiety, schizophrenia, autism, etc., possibly sharing a bidirectional relationship [6,7]. Epilepsy is a disease where people at risk can be identified but nothing can be done to halt or prevent the disease progression [8]. Despite the availability of around 30 United States Food and Drug Administration (USFDA) approved anti-epileptic drugs (AEDs) [9], these drugs only provide symptomatic relief rather than halting/terminating the disease progression. This clearly reflects the complex pathology of epilepsy, reflecting the further extensive need for pre-clinical and clinical research.

Though the precise cause of epilepsy is still elusive, seizures might be the consequence of any insult that disturbs the normal brain function. These insults comprise of acquired causes (stroke or traumatic brain injury), infectious (such as neurocysticercosis), and autoimmune diseases, as well as genetic mutations, etc. [2]. There is an increased understanding of the contribution of neuroinflammation, channelopathies, neurodegeneration, neurogenesis, neural reorganization, and plasticity in epilepsy [10,11]. In recent years, several findings have repeatedly reinforced the role of neuroinflammation in epilepsy [7,12,13], indicating that targeting brain inflammation might be a possible therapeutic strategy against epilepsy. Similarly, traumatic brain injury (TBI) also leads to post traumatic epilepsy (PTE) [14,15]. However, the time duration that the TBI leads to PTE is not well understood, with different existing opinions regarding the percentage that develop epilepsy after TBI. Epilepsy also exhibits idiopathic “*genetic*” etiology or symptomatic “*acquired*” elements. Several susceptibility genes encoding ion channels, including voltage-gated sodium, potassium, and calcium channels, have been unraveled from genetic investigations [11]. Mutations in three alpha subunit genes (*SCN1A*, *SCN2A*, *SCN8A*) of the voltage-gated sodium channels (VGSCs) have been implicated in epilepsy [16]. Voltage-gated potassium (Kv) channels, calcium-activated potassium channels, inwardly rectifying (Kir) channels, and tandem pore domain (K2P) channels have also been implicated in epilepsy [17]. The high-voltage-activated (HVA) Ca_v2.1_ (P/Q-type) channel, encoded by *CACNA1A*, has been associated with early onset epileptic encephalopathy [18].

Neurodegeneration taking place near the epileptogenic regions may induce neuroinflammatory response, network re-organization, and/or a series of molecular changes that may contribute to the transformation of the normal brain to an epileptic state, i.e., temporal lobe epilepsy (TLE) [19]. During epileptogenic phenomena, neurodegeneration mainly occurs in the hilus, cornu ammonis (CA)1, CA2 and CA3 pyramidal cell layer, and granule cells. Besides the hippocampus, neurodegeneration also occurs in the amygdala; the neighboring entorhinal, perirhinal, and para hippocampal cortices; the thalamus; and the cerebellum [20]. Neuroinflammation and excitotoxicity can result in neuronal loss [21], ultimately leading to changes in the hippocampal networks that account for epileptogenesis [22], further suggesting that the manipulation of neurodegenerative phenomena by inhibition of inflammation and excitotoxicity may limit the disruption of the hippocampal circuitry and the progression of epileptic seizures [23]. Moreover, there is evidence that neurodegenerative diseases and epileptogenesis after an acquired brain insult might share a common underlying mechanism [24].

Here, we explore the potential association of the neurodegenerative proteins α-synuclein, beta-amyloid (Aβ), and tau with epilepsy, based on previous clinical and pre-clinical experimental studies [25,26,27,28,29,30], and highlight a novel avenue for research with potential therapeutic targets.

## 2. Alpha-Synuclein-Mediated Neurodegeneration: Implication in Epileptogenesis

The α-synuclein protein is highly charged (residues 120–140) and is located near the synaptic vesicles in the presynaptic terminals [31]. It contributes to the modulation of synaptic transmission, the density of synaptic vesicles, and neuronal plasticity [32,33,34,35]. α-synuclein contributes to several steps of synaptic vesicle recycling, including trafficking, docking, fusion, and further recycling after exocytosis [36]. α-synuclein regulates the size of distinct pools of synaptic vesicles in the mature neurons and reduces the distal reserve synaptic vesicle pool by half with the intact docked vesicle pool [34]. It controls the trafficking of the synaptic vesicles in the distal reserve pool and regulates the amount of vesicles docked at the synapses for neurotransmitter release [37].

In addition, α-synuclein contributes to the folding/refolding of soluble N-ethylmaleimide-sensitive factor attachment protein receptor (SNARE) proteins, which are crucial for the release of neurotransmitters, vesicle recycling, and synaptic integrity [38]. The disruption, aggregation, and deposition of α-synuclein has been implicated as a common phenomenon in neurodegenerative disorders, known as synucleinopathies [39]. However, the underlying mechanism of neurodegeneration caused by the accumulation of α-synuclein is still elusive. α-synuclein exhibits the potential for aggregation and post-translational modifications (PTMs), which may be the cause of its toxic effects [40].

It also interacts with several other proteins associated to neurodegeneration, including tau, transactive response DNA binding protein 43 kDa (TDP-43), Aβ, and prion protein [41]. Apart from synucleinopathies, extracellular α-synuclein also exerts a crucial role in neuroinflammation, neurotoxicity, and in spreading pathology. It is transported to the extracellular milieu after being actively secreted and released from dying neurons. The precise mechanism of secretion of α-synuclein is not well understood. However, an earlier study reported that it can be physiologically secreted by enteric neurons through conventional endoplasmic reticulum/Golgi-dependent exocytosis, in neuronal activity-driven phenomena [42].

In the extracellular space, α-synuclein can initiate the activation of neighboring astrocytes and microglia, enhancing glial proinflammatory activity. Microglial activation in turn produces proinflammatory cytokines, nitric oxide (NO), and reactive oxygen species (ROS), which may be toxic to neurons. α-synuclein can also be transmitted across neurons to initiate aggregation events, leading to compromised viability of the inheritor neuron [39].

Extracellular α-synuclein has also gained increasing attention due to its possible role in disease initiation and progression as evident by the presence of α-synuclein-positive Lewy-body-like insertions in the long-term mesencephalic transplants of Parkinson’s disease (PD) patients [43,44]. Therefore, manipulation of α-synuclein secretion or its neuronal uptake may exert beneficial effects for halting or retarding the spreading of α-synuclein pathology, mainly in PD and in other synucleinopathies.

Despite the known role of α-synuclein in neurodegeneration, limited studies exist on the impact of α-synuclein-mediated neurodegeneration in epileptogenesis. However, emerging data highlight the correlation of α-synuclein with epileptic seizures.

Pentylenetetrazol (PTZ) is a pro-convulsant used to model experimental epilepsy [45,46], where it induces epileptic seizures mainly via binding to the γ-aminobutyric acid (GABA_A_) receptor and hindering the neuroinhibitory potential of GABA [47]. In a PTZ (50 mg/kg, I.P.) kindling-induced experimental epilepsy model of male Sprague-Dawley (SD) rats, upregulated expression of α-synuclein was observed in the hippocampal CA3 area. Moreover, brain sections demonstrated low cytoplasmic expression of α-synuclein in the normal brain and high cytoplasmic expression in the epileptic brain. In addition, its expression was positively correlated with the chronic phase of the seizure score [26]. However, the study did not precisely unravel the role of α-synuclein in generating epileptic seizures. Nevertheless, this finding advocate that α-synuclein might represent a potential target against the development of epileptic seizures.

Pilocarpine is a potent muscarinic receptor agonist [48] used to induce chronic seizure-like conditions [49,50], where the underlying mechanism depends on activation of the muscarinic receptor (M1) and seizures are further maintained by N-methyl-d-aspartate (NMDA) receptors’ activation [51]. Status epilepticus (SE) is defined as a constant or sporadic seizure that lasts for more than 5 min, without a complete recovery of consciousness between seizures [52]. SE can have long-term outcomes, including neuronal death, neuronal injury, and disruption of the neuronal networks, that are dependent on the seizure type and duration [53]. Pilocarpine-induced epilepsy is one of the extensively used models to resemble SE and can exhibit a clinical phenotype of human TLE, such as limbic seizures, secondary generalized seizures, hippocampal sclerosis, mossy fiber sprouting, and SE lasting for several hours [54,55,56]. Protein profiling of the epileptic dentate gyrus (DG) (4 weeks after induction of SE) revealed increased α-synuclein expression by 12.8% in the pilocarpine (325 mg/kg, I.P.)-induced TLE in male C57Bl/6 mice. Moreover, immunohistochemical analysis demonstrated an elevated expression of α-synuclein within the supra-granule region of the DG, confirming α-synuclein dysregulation [57].

In addition to the experimental studies, several clinical data confirm upregulated α-synuclein expression in epileptic patients, strengthening the possibility of α-synuclein-mediated neurodegeneration in epilepsy. Abnormal deposits of the protein have also been observed in the hippocampal specimens obtained from patients with Mesial temporal lobe epilepsy (MTLE) and were associated with the loss of neuronal cells and reactive gliosis in the hippocampus [58].

Pediatric epilepsy is another persistent brain disorder that exhibits behavioral and cognitive challenges, including intellectual dysfunctions, behavioral problems, and attention deficits [25,59]. In a clinical study of children with epilepsy, upregulated expression of serum α-synuclein was observed, with a positive correlation to the measures of disease severity, suggesting that it may present a plausible prognostic biomarker of the neurodegenerative process [25]. In addition, serum α-synuclein levels were positively correlated to exosome α-synuclein levels, the presumptive fraction of the cerebrospinal fluid (CSF). Interestingly, in children with epilepsy, there was a significant correlation of exosomal α-synuclein with serum interleukin (IL)-6 levels, reflecting that exosomal α-synuclein may contribute to the neurotoxic cycle of neuroinflammation in epilepsy patients [25] based on its ability to directly activate the astrocytic production of IL-6 [60].

Although the prognosis of seizure control by the currently available AEDs is good in the majority of patients, one third of them are refractory to the AEDs [61], denoting an intractable epilepsy. α-synuclein may serve as a biological marker in both epilepsy and intractable epilepsy, since an upregulation in its concentration (serum and CSF) has been observed in epileptic patients when compared to normal controls. However, upon subgroup analysis, the level of α-synuclein (serum and CSF) was upregulated in patients with intractable epilepsy while no association was observed in the α-synuclein levels among patients with newly diagnosed epilepsy and non-intractable epilepsy [62], suggesting that the estimation of α-synuclein rates may act as a useful prognostic marker for clinical assessment in intractable epilepsy [62].

Despite the elevation of α-synuclein expression in epilepsy, the precise mechanism underlying the contribution of α-synuclein to the disease pathogenesis is still elusive. In addition, more investigations are warranted to determine whether inhibition of the aggregation of α-synuclein in epilepsy is beneficial. Elucidation of the underlying mechanism behind its secretion and cytotoxicity might open novel perspectives in deciphering the α-synuclein-mediated neurodegeneration in epileptogenesis.

## 3. Aβ-Mediated Neurodegeneration and Its Implication in Epilepsy

The β- and γ-secretase cleavage of amyloid precursor protein (APP) generates Aβ, which is the key component of senile plaques and, along with abnormal Aβ accumulation, represents the hallmark of Alzheimer’s disease (AD). APP is a membrane-spanning protein exhibiting a large extracellular domain and a smaller intracellular domain. APP is acknowledged as the key source of the Aβ peptide observed in the neuritic plaques of AD patients and is a functionally crucial molecule in its full-length configuration, as well as being the source of several fragments with variable effects on neural function [63]. APP was shown to exert crucial physiological roles in the mammalian brain mainly by regulating the synaptic functions and neuronal survival, and even modulating GABA neurotransmission [64]. APP is extensively investigated against AD pathogenesis due to its role in the disease’s pathogenesis through the generation of toxic Aβ aggregates, potentially initiating neurodegeneration [65].

Progressive neurodegeneration, with subsequent cognitive and behavioral impairments, characterizes AD pathogenesis. Aβ aggregation into oligomers and eventually into fibrils is established as the driving mechanism for neurotoxicity [66]. Brain Aβ oligomers, rather than amyloid plaques, are highly associated with neuronal loss [67]. Aβ exists in two different isoforms, with Aβ40 being more abundant, whereas Aβ42 is more susceptible to aggregation and more relevant to the pathogenic process [68]. Intracerebroventricular (ICV) injection of Aβ_1-42_ has been shown to mediate neurodegeneration and induce an AD-like phenotype in animals and non-human primates [69,70]. The underlying mechanisms behind Aβ_1-42_-induced neurodegeneration includes mitochondrial disruption, oxidative stress, degeneration of cholinergic neurons, and increased Aβ_1-42_ deposition, ultimately leading to cell death [71]. Furthermore, Aβ regulates the NMDA receptors (NMDARs) and disrupts the ionic balance between synaptic and extra synaptic NMDAR signaling [72,73]; however, the precise mechanism behind Aβ-mediated excitotoxicity stills remains obscure.

A plethora of data supports Aβ-mediated neurodegeneration in AD [74,75,76]. However, this neurodegenerative protein has also gained increased attention in epileptogenesis. Aβ has mainly been implicated in the pathology of acquired epilepsy, where increased amyloid production and deposition have been shown to contribute to acquired epilepsy [77]. In this case, spontaneous seizures are mainly initiated after injury to the normal brain as a result of brain trauma, stroke, infection, or SE [78]. Tg2576 mice expressing human APP with the Swedish mutation (*K670N/M671L*) guided by the hamster prion protein demonstrated electrically evoked seizures, as evident by the lower after-discharge threshold (ADT) current and increased vulnerability to kindling [77]. In accordance, Tg2576 mice exhibited spontaneous seizures, increased mortality, and lower thresholds to PTZ-induced seizures, suggesting that overexpression of APP might contribute to seizure activity in neurodegenerative disorders [79]. On the contrary, zebrafish larvae lacking APP are susceptible to PTZ-induced seizures. Moreover, it was unraveled that intact prion protein is required for the seizure susceptibility of APP mutants [80].

Investigating the role of Aβ in the context of epilepsy is of crucial importance based on several studies supporting a close association of AD and epileptic seizures, possibly sharing common underlying mechanisms [81,82,83]. In this respect, transgenic mice overexpressing mutant APP and producing excessive amounts of Aβ are crucial for understanding the mechanism of AD pathogenesis [84]. Familial AD (FAD) is the less prominent form of AD, with an earlier onset compared to sporadic AD (accounting for more than 90% of the AD cases). FAD has been associated with mutations in three major genes: *APP*, presenilin1 (*PS-1*), and presenilin 2 (*PS-2*), which ultimately induce an abnormal overproduction of Aβ [85]. *PS-1* is the catalytic subunit of γ-secretase that contributes to the production of Aβ, and gene mutations have a tendency to increase the produced Aβ42/Aβ40 ratio [86]. Moreover, *PS-1* mutations might also cause seizures independent of the APP processing [87]. In a study of *APdE9* mice (carrying mutant human *APPswe* and *PS1dE9* genes), a greater propensity of epileptic seizures was observed at the time of appearance of the first amyloid plaque compared to wild-type (WT) littermates. The Aβ-induced sustained depolarization was proposed as the cause of epileptic seizures in *APdE9* mice [84]. APP metabolites and mainly the APP intracellular domain (AICD) might modulate the neuronal networks as evident by the abnormal electroencephalogram (EEG) spiking events and a strong susceptibility to induced seizures by transgenic mice overexpressing AICD and its binding partner Fe65 [87].

There is further evidence that the neurodegenerative proteins associated with AD are dysregulated during epileptogenesis. In the experimental model of TLE induced by electrical stimulation at an intratrain pulse frequency of 50 Hz in female SD rats, dysregulation in the proteins associated with Aβ processing, deposition, plaque formation, and Aβ-associated pathology was observed from bioinformatics analysis [88].

Pro-epileptogenic effects of Aβ have been reported in the 4-aminopyridine (4AP)-induced seizure model of male Wistar rats. More specifically, a single injection of Aβ was shown to facilitate 4AP-induced seizure expression and decrease the latency for 4AP-induced seizures. It further surged the number of generalized seizures, impaired the time for full recovery, and favored seizure-induced death. These pro-epileptogenic effects of Aβ have been correlated with the disruption of normal hippocampal function by affecting the synaptic efficacy and its coordinated network activity [89].

Kainic acid (KA) is an extensively used epileptogenic, and is the neuroexcitotoxic agent that acts on kainate receptors (KARs) in the central nervous system (CNS) [90]. Systemic administration of KA leads to prolonged seizures, resulting in excitotoxic hippocampal neuronal injury mainly in the CA3 area [91]. In an experimental model of induced TLE in male SD rats by KA (12 mg/kg, I.P.), increased APP expression and its processing enzymes was observed. It is worth noting that APP levels were only increased significantly at 2 and 12 days but not at 12 h post-KA administration when compared to normal control rats. In fact, in the control hippocampus, APP immunoreactivity was mainly located in the CA1-CA3 pyramidal neurons and in granule cells of the DG but not in glial cells. On the contrary, after 12 days of KA administration, APP was localized mainly in the glial cells of the hippocampus. Immunoreactive APP was found to mainly be localized in a subset of glial fibrillary acidic protein (GFAP)-labelled reactive astrocytes. In addition, increased expression of beta-site APP cleaving enzyme 1 (BACE1) and several components of the γ-secretase complex such as presenilin 1 (PS-1), Nicastrin, presenilin enhancer 2 (PEN2), and anterior pharynx defective 1 (APH1), along with elevated expression of Aβ_1-40_ and Aβ_1-42_, was observed in the hippocampus of KA-treated rats compared to normal controls. In accordance, treatment of primary rat astrocytic cultures with KA resulted in increased Aβ production/secretion without compromising the cell viability [27]. This finding suggests that activated astrocytes demonstrate a crucial role in KA-induced neuronal degeneration by upregulating APP expression and increasing Aβ peptide production. Furthermore, it implies that lowering/inhibiting Aβ levels might exert beneficial effects in lessening the seizures and reducing neurodegeneration [27].

The relation between Aβ and epilepsy has also been explored in clinical studies of patients with refractory epilepsy (RE) who had undergone resection of the temporal lobe or hippocampal sections. An increased expression of Aβ precursor protein (β-*APP*) was detected when compared to the controls. Moreover, immunostaining confirmed the localization of β-*APP* mainly in the neuronal cytoplasm and the axons of patients with RE. This finding indicates that elevated β-*APP* expression levels might play a crucial role in the pathomechanism underlying RE [92]. In the hippocampus and temporal lobe cortex of drug-resistant TLE patients who underwent temporal lobe resection, several molecular alterations that resemble those seen in AD patients were observed, including an upregulation of full-length APP expression and enhanced APP amyloidogenic processing, evident by increased phosphorylated APP (pAPP), Aβ_42_, and Aβ_56_ expression [28].

Although there is existing strong evidence that Aβ possibly contributes to the generation of epileptic seizures, and given the availability of several treatment strategies targeting Aβ [93], there is a lack of clinical studies targeting Aβ in epilepsy and therefore the therapeutic value of this intervention remains unanswered.

## 4. Tau-Mediated Neurodegeneration in Epilepsy

Tau is a microtubule-associated protein (MAP) of 440 full-length amino acids with a well-established biological role in microtubule assembly and stability, axonal transport, and neurite outgrowth [94,95]. The tau protein is encoded by the microtubule-associated protein tau *(MAPT)* gene on chromosome 17q21 and contains six splicing isoforms [96]. Tau protein is highly water soluble and structurally unfolded with minimum tendency for aggregation. Tau aggregation into paired helical filaments (PHFs) and neurofibrillary tangles (NFTs) presents the characteristic pathogenic factor of several neurodegenerative diseases known as tauopathies [97]. Interestingly, tau is required to mediate Aβ-induced toxicity [98].

PTMs have been shown to modify tau oligomerization, aggregation, and tau-mediated neurodegeneration. Tau has been reported to undergo several PTMs, including glycosylation, glycation, deamidation, isomerization, nitration, methylation, ubiquitylation, sumoylation, and truncation [99]. They all play a plausible role in regulating tau hyperphosphorylation, which may cause missorting of tau from axons to the somatodendritic compartment, leading to synaptic disruption, as well as induction of its degradation and truncation by proteases. Moreover, tau phosphorylation may enhance its aggregation and change the interaction of tau with its binding partners [97].

Protein phosphatase 2A (PP2A) is known as the main regulator of tau phosphorylation, accounting for around 70% of the total tau phosphatase activity in the human brain [100]. PP2A can regulate tau phosphorylation directly as well as the protein kinases that can phosphorylate tau, including Ca^2+^/calmodulin-dependent protein kinase II (CaMKII), cyclic-AMP-dependent kinase (PKA), cyclin-dependent-like kinase-5 (CDK5), and glycogen synthase kinase 3 (GSK-3β) [101]. Abnormal tau hyperphosphorylation is the crucial step in neurofibrillary degeneration in AD as well as in other tauopathies, where the PP2A activity is compromised. Inhibition of protein kinases that can phosphorylate tau and the improvement of PP2A activity might therefore represent potential therapeutic targets.

Tau protein has been extensively studied in the context of several neurodegenerative disorders, including AD [102,103], frontotemporal dementia (FTD) [104,105], and related tauopathies, including progressive supranuclear palsy (PSP), corticobasal syndrome (CBS), Down’s syndrome (DS), PD, and dementia with Lewy bodies (DLB) [106]. There is a growing interest in investigating tau protein against epilepsy and emerging data indicate tau as a possible target in epilepsy [30].

Tau hyperphosphorylation and aggregation has been observed in intra-amygdala KA-induced TLE in male C57BL6 rats. KA-induced SE resulted in upregulation of total tau levels in the hippocampus mainly in the DG, and in phosphorylation of the AT8 epitope (Ser202, Thr205), mainly in the mossy fibers of the DG [29]. This aberrant tau hyperphosphorylation observed in epileptic mice sheds light on the impact of tau pathology in TLE with significant implications in epilepsy.

There was a two-phase change in the dynamics of tau phosphorylation, an initial dephosphorylation followed by sustained hyperphosphorylation as observed in a KA (20 mg/kg, I.P.)-induced experimental model of excitotoxic damage in male FVB mice. Moreover, tau kinases, such as GSK3β and CDK5, were activated at the first phase (within 6 h post-injection), where GSK3β gradually returned to its basal activity, but CDK5 was further activated during the second phase (after 6 h post-injection). It is worth noting that the study evaluated the dynamics of tau phosphorylation up to 48 h post-KA administration. This data suggests that GSK-3β might not play a role in tau phosphorylation during excitotoxicity, whereas the significant activation of CDK5 during the second phase might contribute to enhanced tau phosphorylation. Moreover, increased PP2A activity during the first phase of KA-induced excitotoxicity when tau was dephosphorylated, speculates that the activation of PP2A contributes to tau dephosphorylation at the initial phase [107].

KA-induced excitotoxicity results in the hyperphosphorylation of tau protein, leading to intracellular inclusion of tau. KA (30 mg/kg, I.P.) administration leads to increased tau phosphorylation at several sites (3 days post-KA injection) (Thr231, Ser199, and Ser396), reflecting destruction of the hippocampal neuronal cytoskeleton in male CD10 mice. KA-induced SE further reduced protein kinase B (AKT) activity (1–3 days after KA administration) and activated tau kinases, GSK3β and CDK5 (higher at 3 days post-KA treatment and gradually decreased at 7 days after KA injection) [108]. In an experimental model of SE induced by KA (15 mg/kg, I.P.) in adult male C57BL6 mice, the protein levels of tau kinase GSK3β were highly expressed in the ipsilateral hippocampus after SE. Moreover, overexpression of GSK3β aggravated the seizure-induced cell death as evident by the elevation in the seizure-induced hippocampal neuronal death of mice overexpressing GSK3β [109].

Furthermore, tau hyperphosphorylation was observed in a clinical study as well. In addition, CSF tau has been suggested as a plausible biomarker for the prediction of neuronal damage after epileptic seizures [110]. Hyperphosphorylated tau protein identified by immunohistochemistry (using AT8 staining) has mainly been detected in the form of neuropil threads, NFTs, and pre-tangles within the temporal lobe tissue of TLE patients who have undergone anterior temporal lobe resection [111]. As observed in AD [112], TLE patients who underwent anterior temporal lobe resections demonstrated significant tau pathology, as evident by the significant upregulation of tau 5 expression in the TLE hippocampus and not in the temporal cortex. Moreover, phospho-Tau AT8 expression was upregulated significantly in both the TLE hippocampus and temporal cortex [28], revealing an interaction between hyperexcitability in TLE and tau-related neurodegeneration. However, the detailed mechanism underlying tau release during TLE is still obscure, but the detection of tau pathology may provide evidence of disease progression in TLE [113].

Given the implication of tau pathology in epilepsy, with its hyperphosphorylation being suggested to promote vulnerability to epileptogenesis [114], tau protein might represent a putative therapeutic target in preventing epilepsy. Sodium selenate, a potent activator of PP2A/PR55 [115] that enhances PP2A activity by stabilizing the protein phosphatase, has demonstrated anti-epileptogenic effects against chronic acquired epilepsy induced by amygdala kindling and in post-KA-induced SE in male Wistar rats. Sodium selenate treatment delayed the amygdala kindling epileptogenesis as evidenced by the slow progression of seizures and an increased number of stimulations required to reach several kindling stages. Likewise, the anti-epileptogenic effects of sodium selenate against post-KA-induced SE are evident by the decreased number of seizures and their duration per day on video-EEG monitoring while they are mainly mediated by stimulation of PP2A and reduction on the levels of hyperphosphorylated tau. More specifically, treatment with sodium selenate for 4 and 8 weeks in the amygdala kindling and in the post-KA-induced SE model, respectively, attenuated the chronic epilepsy-induced reduction in PP2A activity and PR55 levels as well as reducing phospho-Tau levels [30]. Moreover, sodium selenate exerted protection against 6-Hz electrical stimulation-induced seizure (acute treatment) in male CF albino mice, whereas chronic treatment with sodium selenate reduced the seizure number induced by PTZ injection, resembling its anti-epileptic effects [116]. Antisense oligonucleotides were shown to protect against picrotoxin-induced hyperexcitability and PTZ-induced seizures based on endogenous tau mRNA and protein expression analysis, suggesting that antisense oligonucleotides’ reduction of tau may prove beneficial in epileptic seizures [117].

Although the targeting of hyperphosphorylated tau demonstrated promising beneficial effects against epileptic seizures as discussed above, different data have been reported regarding GSK3β, a kinase that is involved in tau phosphorylation. GSK-3 inhibitors (NP031112 and NP060103) have been demonstrated to exacerbate hippocampal damage and increase seizure severity during KA-induced SE, warranting caution against targeting GSK-3β in epilepsy [109].

Altogether, although hyperphosphorylated tau has been implicated in epilepsy, it is worth noting that unmodified tau protein is not required for epileptogenesis but rather PTMs of tau protein build a pathologic environment that assists epileptogenesis. This supports the notion that inhibition of abnormal tau pathology leading to the prevention of accumulation of hyperphosphorylated tau might represent a promising anti-epileptogenic strategy [114].

## 5. Discussion

Despite the extensive clinical and pre-clinical studies aiming to elucidate the mechanisms underlying epileptogenesis, there are still no pharmacological strategies to prevent it or modify its progression. The available AEDs provide only symptomatic relief whereas one-third of the epileptic patients develop drug resistance [61], indicating the high demand for novel therapeutic strategies that can modify the disease progression.

In recent days, there has been a growing interest in exploring the contribution of neurodegenerative pathways in epilepsy, focusing in particular on α-synuclein, Aβ, and tau. α-synuclein has been widely explored in PD and other synucleinopathies [118,119,120], where its aggregation impairs neuronal, synaptic, and mitochondrial functions along with protein degradation pathways, ensuring its role in PD pathogenesis [121]. In addition, Aβ and tau have been implicated in neurodegenerative disorders, mainly AD, where the accumulation of Aβ initiates an insult that ultimately drives the accretion of tau pathology and tau-mediated neurodegeneration [122].

Upregulated α-synuclein levels have been reported in several pre-clinical and clinical epilepsy studies (Table 1). However, the precise mechanism behind the contribution of α-synuclein in epilepsy remains obscure. The AD-like pathology, as evident by upregulated levels of Aβ and tau in TLE, suggests that these neurodegenerative pathways play a role in TLE [28]. Elevated expression of APP and its processing enzymes, β-*APP* expression, and Aβ_1-40_ and Aβ_1-42_ levels in experimental and human epilepsy (Table 2) suggest a possible role in mediating neurodegeneration in epilepsy. At the same time, increased tau hyperphosphorylation has been detected in human epilepsy and animal models (Table 3). Although the underlying mechanism of tau-mediated neurodegeneration in epilepsy is not completely understood, there is evidence that tau contributes to epileptogenesis, mainly by reducing PP2A activity, increasing the activity of GSK3β and CDK5, activating the PI3K/AKT pathways, promoting the NMDAR subtype 2B (NR2B) receptor-mediated glutamate release, and stimulating NMDAR [123].

All the above findings support the role of α-synuclein, Aβ, and tau in epilepsy and indicate that these neurodegenerative proteins may present therapeutic targets in epilepsy. Having said that, neurodegeneration is a progressive phenomenon that occurs during acquired epileptogenesis [124], with α-synuclein, Aβ, and tau constituting its primal mediators. It is worth mentioning here that neurodegeneration might not directly lead to epileptogenesis, but neurodegeneration might prompt other processes to initiate epileptogenesis [125,126]. Moreover, the precise association between neurodegeneration and epileptic seizures is not fully understood [24]. However, it could be possible that the neurodegeneration detected in patients and in experimental models with acquired epilepsy is the consequence of an injury or a secondary effect of repeated epileptic seizures [127,128].

Summing up, the present review greatly advances our knowledge about the plausible role of α-synuclein, Aβ, and tau in epileptogenesis, providing a novel perspective. Nevertheless, the evidence discussed herein comes mainly from pre-clinical studies, and extensive research is warranted for the clinical translation of these findings.

## Figures and Tables

**Table 1:** Summary of studies investigating α-synuclein against epilepsy.

S.N.	Study Type	Epilepsy Model	Observations	References
1	Experimental study	PTZ (50 mg/kg, I.P)-induced epilepsy in male SD rats	PTZ administration leads to upregulated mean region of interest of α-synuclein positive cells in the hippocampal area. α-synuclein expression has been correlated with the seizure score.	[26]
2	Experimental study	Pilocarpine (325 mg/kg, I.P.)-induced TLE in male C57Bl/6 mice	Immunohistochemical analysis showed upregulation of α-synuclein within the supra-granule region of the DG from Pilocarpine-induced TLE rats. Western blot analysis reported that TLE triggered an increase in α-synuclein expression by 12.8%.	[57]
3	Clinical study	Children with epilepsy (*n* = 115) and normal controls (*n* = 146)	Serum α-synuclein levels were increased significantly in children with epilepsy and was correlated with disease severity. Exosome α-synuclein levels were correlated with serum α-synuclein.	[25]
4	Clinical study	Epileptic patients (*n* = 67) with three subtypes: Patients with intractable epilepsy (*n* = 40); patients with newly diagnosed epilepsy (*n* = 13); patients with non-intractable epilepsy (*n* = 14)	There was significant elevation in the concentration of α-synuclein (serum and CSF) in the epileptic patients when compared to normal. Levels of α-synuclein (serum and CSF) were increased in patients with intractable epilepsy whereas there was no difference in α-synuclein levels between patients with newly diagnosed epilepsy and non-intractable epileptic patients.	[62]
5	Human specimens	Hippocampal specimens obtained at surgery from patients with MTLE	Altered expression of α-synuclein was obtained in the human hippocampus.	[58]

PTZ, Pentylenetetrazol; TLE, Temporal lobe epilepsy; SD, Sprague-Dawley; I.P., Intraperitoneal; DG, Dentate gyrus; MTLE, Mesial temporal lobe epilepsy; CSF, Cerebrospinal fluid.

**Table 2:** Summary of study investigating Amyloid β in epilepsy.

S.N.	Study Type	Study Design	Observations	References
1	Experimental study	KA (12 mg/kg, I.P.) induced TLE in male SD rats	Increased level and expression of APP and its processing enzymes (BACE1, PS1, Nicastrin, PEN2, APH1) is observed in KA-induced rats. As detected by ELISA, Aβ_1-40_ and Aβ_1-42_ levels are increased in the hippocampus of KA-treated rats compared to normal controls.	[27]
2	Experimental study	TLE induced by electric stimulation in female SD rats where the SE was confirmed by EEG recordings	Epileptogenesis-related dysregulation of proteins involved in Aβ processing and its regulation was observed. Dysregulation in APP and α-secretase, α-disintegrin metalloproteinase was observed.	[88]
3	Clinical study	TLE group with RE who underwent anterior temporal lobe resections (*n* = 19) and normal controls (*n* = 22)	Significant elevation in full-length APP expression was observed in the TLE hippocampus but not in TLE cortex. Upregulation in APP amyloidogenic processing has been observed in TLE patients as evident by increased pAPP, Aβ_42_ and Aβ_56._	[28]
4	Clinical study	Tissue samples obtained from patients with RE (*n* = 36)	β-*APP* expression was increased in patients with RE as compared to the normal controls. The β-*APP* protein was mainly localized in the neuronal cytoplasm and axons of epileptic patients.	[92]

KA, Kainic acid; Temporal lobe epilepsy; SE, Status Epilepticus; EEG, Electroencephalogram; SD, Sprague-Dawley; RE, Refractory epilepsy; APP, Amyloid precursor protein; Aβ, Amyloid-Beta; ELISA, Enzyme-linked immunosorbent assay; BACE1, Beta-site amyloid precursor protein cleaving enzyme 1.

**Table 3:** List of studies investigating tau protein in epilepsy.

S.N.	Study Type	Study Design	Observations	References
1	Experimental study	Intra-amygdala KA-induced SE in male C57BL6 rats	There was an upregulation of total tau levels and tau phosphorylation in the hippocampus post-SE. There was an elevation in tau phosphorylation during epilepsy at the AT8 epitope; however total tau expression was decreased in the hippocampus mainly in the CA3 and CA1 subfield.	[29]
2	Experimental study	KA (15 mg/kg, I.P.)-induced SE in male C57BL6 rats	There was a significant increase in the protein expression of tau kinase GSK3β in the ipsilateral hippocampus after SE.	[109]
3	Experimental study	Chronic acquired epilepsy induced by amygdala kindling and KA in male Wistar rats	Treatment with Sodium selenate decreased activity of PP2A, increased ratio of pS198 and pS262 immunoreactivity to tau-5 was observed in amygdala, hippocampus and cortex of both amygdala kindled and KA-induced SE rats. T-Tau levels remained uninfluenced in both the models.	[30]
4	Experimental study	KA (20 mg/kg, I.P.)-induced excitotoxicity damage in Male FVB mice	KA-induced excitotoxic damage leads to short-term tau hypophosphorylation followed by a gradual long-term hyperphosphorylation of tau. The initial dephosphorylation of tau in the first phase (within 6 h post-injection) might be due to PP2A activation and the gradual hyperphosphorylation of tau at later phase (after 6h post-injection) could be mainly due to CD5K activation and inhibition of PP2A during the second phase.	[107]
5	Experimental study	KA (30 mg/kg, I.P.)- induced SE in male CD10 mice	KA-induced SE leads to tau hyperphosphorylation, which might be due to increased activity of tau kinase (GSK3β, CDK5) and inactivation of AKT.	[108]
6	Clinical study	Drug-resistant TLE patients who had undergone anterior temporal lobe resection (*n* = 19)	Upregulation in the expression of tau 5 was observed in the TLE hippocampus but not in the temporal cortex. However, phospho-Tau AT180 was increased in both the hippocampus and temporal cortex of TLE patients. Among the tau isoforms containing 3 (3R) or 4 (4R) microtubules binding repeats, tau 3R expression was unaltered, but tau 4R expression was increased in TLE patients compared to the normal controls.	[28]
7	Clinical study	Patients TLE who had undergone anterior temporal lobe resection (*n* = 33)	Hyperphosphorylated tau (AT8 labelling) was mainly observed in the form of neuropil threads, NFTs and pre-tangles within the temporal lobe tissue. 31 out of 33 TLE patients exhibited AT8 labelling.	[111]
8	Clinical study	Patients with tonic-clonic or partial secondarily generalized seizures are considered (*n* = 54)	The median T-Tau and p-Tau was 163.1 pg/mL and 39.6 pg/mL respectively in the patients whereas for the control the value of T-Tau and p-Tau was 143.5 pg/mL and 38.1 pg/mL respectively. However, there was no significance difference between the groups. There was significant difference between ration of T-Tau/p-Tau between epileptic and control group.	[110]

KA, Kainic acid; TLE, Temporal lobe epilepsy; SE, SE, Status Epilepticus; GSK3β, Glycogen synthase kinase-3β; PP2A, Protein phosphatase 2A; AKT, Protein kinase B; CDK5, Cyclin-dependent kinase 5; NFTs, Neurofibrillary tangles; CA3, Cornu Ammonis 3; CA1, Cornu Ammonis 1; T-Tau, Total tau; P-Tau, Phosphorylated tau.

## Abbreviations

Aβ	Amyloid β
AD	Alzheimer’s disease
NFTs	Neurofibrillary tangles
MAP	Microtubule-associated protein
APP	amyloid precursor protein
TLE	temporal lobe epilepsy
KA	Kainic acid
PTZ	Pentylenetetrazol
CDK5	Cyclin dependent kinase 5
GSK3β	Glycogen synthase kinase 3β
PP2A	protein phosphatase 2A
PI3K	Phosphatidylinositol 3-kinase
CaMKII	Ca^2+^/calmodulin-dependent protein kinase II
PHFs	Paired helical filaments
FTLD	Frontotemporal lobar degeneration
PTM	Post-translational modification
CA	Cornu Ammonis
TDP-43	Transactive response DNA binding protein 43 kDa
ROS	Reactive oxygen species
SD	Sprague-Dawley
CSF	Cerebrospinal fluids
NMDARs	N-methyl-D-aspartate receptors
GFAP	Glial fibrillary acidic protein
